# Coronary Artery Calcification by Computed Tomography in Epidemiologic Research and Cardiovascular Disease Prevention

**DOI:** 10.2188/jea.JE20110138

**Published:** 2012-05-05

**Authors:** Akira Sekikawa, J. David Curb, Daniel Edmundowicz, Tomonori Okamura, Jina Choo, Akira Fujiyoshi, Kamal Masaki, Katsuyuki Miura, Lewis H. Kuller, Chol Shin, Hirotsugu Ueshima

**Affiliations:** 1 Department of Epidemiology, Graduate School of Public Health, University of Pittsburgh, Pittsburgh, PA, USA; 2 Department of Geriatric Medicine, the John A. Burns School of Medicine, University of Hawaii, Honolulu, HI, USA; 3 UPMC Heart and Vascular Institute, University of Pittsburgh Medical Center, Pittsburgh, PA, USA; 4 Department of Preventive Medicine and Public Health, School of Medicine, Keio University, Tokyo, Japan; 5 Department of Community Health Nursing, College of Nursing, Korea University, Seoul, Korea; 6 Department of Health Science, Shiga University of Medical Science, Otsu, Japan; 7 Department of Internal Medicine, Korea University, Ansan, Korea

**Keywords:** coronary artery calcification, primary prevention, coronary calcium score, EBCT, MDCT

## Abstract

Both American and European guidelines recommend coronary artery calcification (CAC) as a tool for screening asymptomatic individuals at intermediate risk for coronary heart disease (CHD). These recommendations are based on epidemiologic studies mostly in the United States. We review (1) the use of CAC in primary prevention of CHD in the United States, (2) epidemiologic studies of CAC in asymptomatic adults outside of the United States, and (3) international epidemiologic studies of CAC. This review will not consider clinical studies of CAC among patients or symptomatic individuals. US studies have shown that CAC is a strong independent predictor of CHD in both sexes among middle-aged and old age groups, various ethnic groups, and individuals with and without diabetes and that CAC plays an important role in reclassifying individuals from intermediate to high risk. Studies in Europe support these conclusions. The Electron-Beam Tomography, Risk Factor Assessment Among Japanese and US Men in the Post-World-War-II birth cohort (ERA JUMP) Study is the first international study to compare subclinical atherosclerosis, including CAC among Japanese, Japanese Americans, Koreans, and whites. It showed that as compared with whites, Japanese had lower levels of atherosclerosis, whereas Japanese Americans had similar or higher levels. CAC is being increasingly used as a screening tool for asymptomatic individuals in Europe and the United States. CAC is a powerful research tool, because it enables us to describe differences in atherosclerotic burden across populations. Such research could identify factors responsible for differences among populations, which may improve CHD prevention.

## INTRODUCTION

Screening asymptomatic individuals for subclinical atherosclerosis is the subject of intensive research.^1^ Histopathologic studies show that the extent of atherosclerosis is associated with traditional risk factors for cardiovascular disease.^2^^,^^3^ However, substantial variation in the extent of atherosclerosis exists at every level of risk factor exposure. The variation is due to genetic susceptibility, combinations and interactions with other risk factors, duration of exposure, and other factors.^4^ Noninvasive measures of subclinical atherosclerosis represent the end result of risk exposure. Thus, they can play an important role in reclassifying individuals from intermediate risk, using a traditional risk prediction model, to high risk. Such reclassification is critical because most cardiovascular events occur in individuals at intermediate risk, and interventions to reduce risk among individuals at high risk are better established than those for individuals at intermediate risk.^1^

Noninvasive measurements of subclinical atherosclerosis include coronary artery calcification (CAC), intima-media thickness (IMT) and plaque of the carotid artery, the ankle-arm index, and aortic pulse wave velocity (PWV).^1^ Although these measurements generally predict cardiovascular events independent of traditional risk factors, the use of these measurements as routine screening tools is controversial.^1^ Clinical guidelines in Europe and the United States recommend CAC as a screening tool for individuals at intermediate risk.^5^^,^^6^ These recommendations are based on the results of epidemiologic studies of CAC in asymptomatic adults and population-based samples, most of which were conducted in the United States.

We review the use of CAC in primary prevention in the United States. Then, we review epidemiologic data on CAC in asymptomatic adults and population-based samples from studies conducted mostly outside of the United States. Finally, we review international epidemiologic studies of CAC, focusing on the Electron-Beam Tomography, Risk Factor Assessment Among Japanese and US Men in the Post-World-War-II Birth Cohort (ERA JUMP Study).^7^ Clinical studies of CAC among patients and symptomatic subjects are outside the scope of this review.

## USE OF CAC IN PRIMARY PREVENTION IN THE UNITED STATES

The extent of atherosclerosis is a powerful determinant of the risk of clinically manifest coronary heart disease (CHD). This was initially evident in studies of patients with suspected angina who had undergone coronary angiography before the development and wide application of coronary artery bypass surgery.^8^ A more recent meta-analysis of 30 prospective cohort studies showed that the presence of vascular calcification consistently increased the risk of cardiovascular and all-cause mortality.^9^ Budoff et al showed that the presence of calcified plaque in the coronary arteries independently predicted a 1.7-fold increase in mortality and that when CAC was extensive, mortality increased 60-fold.^10^ Individuals with a coronary calcium score (CCS) of 0 are at very low risk of CHD.^11^

Electron beam computed tomography (EBT) and multidetector computer tomography (MDCT) were introduced in 1987 and 1999, respectively, as clinical tools for detecting coronary calcium.^12^ The predictive ability of CAC detected by EBT in asymptomatic individuals was first reported in 1999.^13^ Although EBT technology has not substantially changed over the years, MDCT technology has continued to improve, particularly its temporal and spatial resolution. The sensitivity and specificity of MDCT for calcium detection are now similar to those of EBT. Radiation exposure during MDCT non-contrast calcium scoring is now only slightly higher than that during EBCT technology. With the optimal temporal and spatial resolution of these 2 techniques, even low levels of CAC can be visualized within a few seconds and reproducibly quantified by means of well standardized scores. CAC is defined as a hyperattenuating lesion above a threshold of 130 Hounsfield units (HU) and with an area of at least 3 adjacent pixels.^12^ The most widely used method for CAC quantification is the Agatston score,^14^ although “volume score” and “mass score” have also been proposed as alternatives.^15^^,^^16^

Atherosclerotic vascular calcification results from cellular osteogenic differentiation in response to inflammatory injury. Although radiologic imaging techniques exploit the presence of calcium to identify individuals at risk for clinical events, there is also a plausible mechanistic link to plaque rupture. Calcium deposits likely affect plaque stability by introducing “compliance mismatch” at the interface of rigid mineral with more distensible arterial wall tissue. Under the mechanical stress of the constantly beating heart, this interface has an increased risk of failure leading to plaque rupture.^17^ Importantly, a high CCS represents a high overall atherosclerotic burden, and plaque rupture by any mechanism is more likely to occur when the overall plaque burden is high.

A recent report by the American College of Cardiology/American Heart Association supports MDCT and EBT measurement of CAC as reasonable for asymptomatic adults with an intermediate (10%–20%) 10-year risk, based on the National Cholesterol Education Program Adult Treatment Panel (NCEP ATP III) guideline,^18^ and individuals at low to intermediate (6%–10%) risk, but not for individuals with low (<6%) risk.^1^ CAC extent is a strong and independent predictor of CHD risk in men, women, middle-aged to very old adults, African Americans, whites, and individuals with and without diabetes. The association is continuous across CCS level, probably from a CCS of 10 or even lower. Some investigators suggest that a CCS of 100 or higher indicates high risk and that a CCS of 400 or higher indicates very high risk. As noted previously, individuals with a CCS of 0 are at very low risk of CHD.^11^ Although some CHD events do occur in individuals without coronary calcium, they are rare in the population and may be due to coronary artery spasm, or rupture of an isolated coronary plaque due to unexpected stressors. It has been suggested that a portion of the population may have a high burden of noncalcified atherosclerotic plaques. If so, the number of such individuals must be very low given the extremely low risk of CHD, at least over 5 to 6 years, among individuals with a CCS of 0.^19^^,^^20^

The Multi-Ethnic Study of Atherosclerosis (MESA) in the United States is a study of subclinical atherosclerosis and the risk factors that predict progression to cardiovascular disease or progression of subclinical atherosclerosis in a population-based sample of 6814 white, African-American, Hispanic, and Chinese-American men and women aged 45 to 84 without clinical cardiovascular disease at baseline.^21^ MESA reported that CHD risk by level of CCS was independent of the Framingham risk score (FRS) for both men and women—at least within the intermediate risk level of FRS—for most participants.^22^ They also noted a disconnection between recommendations for lipid-lowering therapy based on NCEP ATP III guidelines and CAC measurements at baseline.^23^ For example, among 663 participants with a CCS of 400 or higher, (ie, individuals at very high risk of clinical CHD), only half met current ATP III criteria for lipid-lowering therapy. In contrast, among 3361 with a CCS of 0 and a very low risk for clinical CHD events, 674 (approximately 20%) were candidates for lipid-lowering therapy based on ATP III guidelines. There is a general consensus that aggressive risk factor modification in individuals with high atherosclerotic plaque burden, as indicated by elevated CCS, will be similarly effective in reducing atherosclerotic events, as has been shown in individuals with clinically established CHD.^24^ However, randomized controlled trials of CHD outcomes resulting from CCS-guided aggressiveness of risk factor modification have not been conducted.^25^

Risk assessment by FRS or CCS will not by itself reduce CHD incidence. Individuals identified for specific pharmacologic or even nonpharmacologic therapy must adhere to long-term therapy if a reduction in risk factor level is to be effective. Poor adherence to therapy may be the most important impediment to a substantial reduction in CHD incidence, irrespective of the method of risk assessment. Recent studies have suggested that adherence has improved after CT scanning, especially among individuals with a high CCS.^26^^,^^27^

## CAC IN ASYMPTOMATIC SUBJECTS AND POPULATION-BASED SAMPLES IN EPIDEMIOLOGIC STUDIES CONDUCTED MAINLY OUTSIDE OF THE UNITED STATES

A relatively small number of epidemiologic studies of CAC in asymptomatic individuals or population-based samples have been conducted outside the United States. This section describes studies in Europe, Asia, the Middle East, and a study of Japanese Americans. Other studies comparing CAC prevalence in South America or Europe with that in the United States will be briefly described in the next section. In almost all these studies, CAC was assessed by using EBT or MDCT and CCS was calculated using the Agatston method.

### Studies in Europe

Two well-designed population-based studies of CAC have been conducted in Europe: the Rotterdam Coronary Calcification Study and the Heinz Nixdorf RECALL Study. The former was a study of 2013 men and women aged 55 to 84 years in Rotterdam, the Netherlands in 1997–2000. Cross-sectional analyses showed that CAC was significantly associated with traditional risk factors,^28^ myocardial infarction^29^ and stroke.^30^ Longitudinal analyses showed that CAC was a predictor of CHD independent of traditional risk factors^31^ and was useful for reclassifying individuals into more appropriate risk categories.^32^ Although these findings are consistent with those from IS studies, this study extends the findings among elderly adults. The Heinz Nixdorf RECALL Study was a prospective study of 4078 men and women aged 45 to 74 years in 3 German cities that investigated the predictive value of CAC for future cardiovascular events.^33^^–^^35^ The study found that CCS significantly improved categorization of individuals into risk groups for cardiovascular events, and more importantly, led to a significant reclassification of individuals in the intermediate-risk group, thus enabling identification of individuals at increased risk for cardiovascular events.^33^

### Studies in Asia

Two studies in Japan and 2 studies in Korea investigated CAC in asymptomatic individuals.^36^^–^^39^ Itani et al reported that CAC prevalence was higher in individuals who died of cardiovascular disease than in individuals who died of other diseases. They screened 6212 participants (3377 men and 2743 women, mean [SD] age of 61.4 [11.3]) for lung cancer by using mobile helical CT in 1996–97. CAC was defined as a CT density greater than 110 HU in 5–7 slices of coronary arteries. During an average follow-up period of 4 years, 14 died of cardiac disease, 10 of whom had CAC, and 64 died of other diseases, 31 of whom had CAC. Ikeda et al reported that, among 1100 adults (865 men and 235 women, mean [SD] age of 53.8 [10.9]) who underwent physical checkups, CAC was not independently associated with cerebral atherosclerosis assessed by magnetic resonance imaging.^36^ CAC was assessed using helical CT in 2001–02. The prevalence of CAC, defined as a CCS greater than 500, was 14.2% for men and 6.5% for women. As compared with individuals without CAC, individuals with CAC tended to be older and were more likely to be current or past smokers and to have diabetes mellitus, hypertension, and hypercholesterolemia.

Two recent studies investigated CAC in asymptomatic individuals in Korea.^38^^,^^39^ In an assessment of 1653 adults (1471 men and 182 women, mean [SD] age of 51 [8]), who were examined during routine health screening and volunteered for CAC evaluation, Sung et al reported that CAC may be clinically useful in older individuals and in individuals with metabolic syndrome. The presence of CAC was associated with male sex, age, pulse pressure, metabolic syndrome, and obesity. A large discrepancy between the presence of CAC and 10-year absolute risk assessed by FRS was observed in older individuals and in individuals with metabolic syndrome. Park et al reported the prevalence and distribution of CAC, among 5239 individuals (3422 men and 1817 women mean [SD] age of 53 [9]) and compared the prevalence with that reported in MESA.^38^ The prevalence of a CAC of 100 or lower, greater than 100 to 400, and greater than 400 was 21.9%, 7.7%, and 3.1%, respectively. As compared with MESA, the 50th percentile of CCS was lower in all age groups among Koreans; the CCSs for Koreans versus those in MESA were 1.1 28, 44 145, and 80 385 in men aged 55 to 64, 65 to 74 and 75 to 84 years, respectively.

### A study in the Middle East

Dakik et al examined 1154 asymptomatic adults (938 men, mean [SD] age of 51.8 [10.6] and 216 women, mean age [SD] of 53.4 [10.8]) in Lebanon in 2002–04,^40^ and found that age, hypercholesterolemia, diabetes, and smoking had significant associations with CAC in men whereas only age and hypercholesterolemia had significant associations with CAC in women.

### A study in Japanese Americans

The Honolulu Heart Program (HHP) reported the prevalence of CAC, as well as risk factors and mortality associated with CAC, in Japanese Americans.^41^ The HHP is a longitudinal study of CHD and stroke in a cohort of Japanese-American men and was initiated in 1965.^42^^,^^43^ Although men in the HHP have had lower rates of CHD than whites, associations of risk factors with CHD are similar between these groups.^44^

A few studies have evaluated the distribution of CAC in elderly populations. The HHP evaluated CAC in 222 men aged 84 to 96 years in 2004–5. Extensive CAC was common, and the findings were comparable to those from the Cardiovascular Health Study.^45^ Only 15% of the sample had a CCS lower than 100, and a CCS greater than 1000 was seen in 32%. After adjusting for age, CAC was significantly and inversely associated with low-density lipoprotein cholesterol (LDL-C) and diastolic blood pressure, but not with smoking, high-density lipoprotein cholesterol (HDL-C), and diabetes. Generally these findings were consistent with previous observations, which showed that associations of traditional risk factors with CHD tend to weaken or change with age, perhaps due to age-related declines in the levels of risk factors, competition from clusters of comorbidities, and survivor bias.^45^^,^^46^ Systolic and diastolic blood pressure and body mass index (BMI) that had been measured 40 years earlier (1965–1968) were significantly related to CAC. A higher CCS was associated with increased 3-year all-cause mortality in elderly men. Mortality rates increased significantly with CCS, from 0 in individuals with a CCS lower than 10, to 13.2/1000 person-years in those with a CCS of 10 to 100, and to 48.6/1000 person years in those with a CCS greater than 1000. This association persisted after adjustment for age and cardiovascular risk factors.

## CAC IN INTERNATIONAL EPIDEMIOLOGIC RESEARCH

Investigation of factors responsible for international variations in the distributions of disease rates and risk factors can increase understanding of genetic and environmental origins of disease, which could result in disease prevention.^47^ Several studies reported international variation in CAC prevalence.^41^^,^^48^^–^^51^ Santos et al compared CAC in 17 563 asymptomatic individuals from Portugal, Brazil, and the United States and concluded that the US population, despite its lower risk profile, had a greater burden of disease than the Portuguese and Brazilian populations. Schmermund et al compared the distribution of CAC and the association of CAC with risk factors between population-based studies in Germany (3120 men and women) and the United States (703 men and women)^48^ and concluded that the prevalence of CAC was similar between the 2 populations after adjusting for risk factors and that risk factors associated with CAC were similar between the 2 studies. These studies used existing datasets to compare CAC and its risk factors. In contrast, the ERA JUMP Study was initiated to compare the extent of subclinical atherosclerosis, including CAC primarily among whites, Japanese, and Japanese Americans. In the next section, we provide an overview of the ERA JUMP Study, including its aims, background, findings, and future directions.

### ERA JUMP Study

The ERA JUMP Study was initiated in 2002 and is the first international epidemiologic study to use standardized methods to assess measures of subclinical atherosclerosis in population-based cohorts.^7^ Primary aims at baseline were: (1) to test the null hypothesis that there is no difference in CAC prevalence and carotid IMT levels among men aged 40 to 49 from 3 populations with different CHD mortality but very similar traditional risk factor profiles (population-based samples of approximately 300 whites from Allegheny County, Pennsylvania, United States, approximately 300 Japanese from Kusatsu City, Shiga, Japan, and approximately 300 Japanese Americans who were recruited from the offspring of HHP members), (2) to determine the relationship of risk factors to CAC and IMT within and across these population samples, and (3) to explore factors responsible for the difference in levels of subclinical atherosclerosis. The ERA JUMP Study has also examined population-based samples of 302 Korean men aged 40 to 49 years in Ansan, South Korea^52^^,^^53^ and 107 African-American men aged 40 to 49 years in Allegheny County, Pennsylvania, United States.^54^

### Background and significance

The Seven Countries Study showed that Japan had the lowest CHD mortality among developed countries. This was largely attributed to low dietary intake of saturated fat and cholesterol, resulting in low serum total cholesterol—165 mg/dL in Japan versus 240 mg/dL in the United States in the 1960s.^55^ The hypothesis at that time was that Western acculturation would increase Japanese CHD rates to the levels in the United States.

After World War II (WWII), dramatic changes in lifestyle took place in Japan. National surveys showed that dietary intake of total fat, animal fat, and animal protein increased dramatically between the 1960s and 1990s.^56^ The current dietary intake of cholesterol in Japan is higher than in the United States (446 and 348 mg/day, respectively),^57^ although dietary intake of saturated fat is still lower in Japan than in the United States, 6/1% (15.6 g/day) in Japan versus 10.8% (32.8 g/day) of total kilocalories in the United States.^57^ Since the 1960s, serum total cholesterol level has steadily increased in Japan,^58^ eg, from 188 to 204 mg/dL for men aged 40 to 49.^59^

There is, however, little evidence that CHD mortality in Japan has increased since the 1960s, despite the fact that serum cholesterol and other traditional risk factors are independently associated with CHD in Japanese. In fact, CHD mortality has been decreasing since 1969.^60^^,^^61^ Additionally there has been little evidence that CHD incidence in men in Japan has increased since the 1960s,^62^^–^^64^ although 2 recent studies suggest such an increase.^65^^,^^66^ The incidence of acute myocardial infarction in men in Japan, ascertained by the criteria of WHO MONICA, is about one-tenth that in the United States.^67^ Recent longitudinal studies in Japan showed that serum total cholesterol, LDL-C, and HDL-C are significantly associated with CHD events in Japan.^63^^,^^68^^–^^74^ Moreover, the relative but not absolute risk of CHD associated with serum total cholesterol among Japanese in Japan is similar to that among white populations.^75^ Similar results were noted for blood pressure and smoking, that is to say, the absolute risks were lower, but the relative risks were similar.^70^^,^^76^^,^^77^

The low CHD mortality among Japanese in Japan is unlikely to be due to genetic factors or some lifestyle specific to Asian populations, and the low CHD mortality among men in the post-WWII birth cohort is unlikely to be due to misclassification of causes of death or a cohort effect. CHD rates increased substantially in Japanese migrants to the United States within 1 or 2 generations.^78^^,^^79^ CHD mortality in Asia is increasing along with a concomitant rise in population levels of serum total cholesterol, except in Japan.^80^^,^^81^ The WHO MONICA Study in Beijing, China showed that CHD mortality in Beijing increased by 50% between 1984 and 1999, and 70% of the increase could be attributed to a rise in serum total cholesterol.^82^ Careful evaluation of CHD mortality showed that low CHD mortality in men in the post-WWII birth cohort in Japan is not due to misclassification of cause of death.^83^ In this birth cohort, levels of serum total cholesterol and blood pressure have been similar throughout their lifetime,^59^^,^^84^^–^^87^ and the prevalence of type 2 diabetes appears to be similar between Japanese in Japan and US whites.^88^^,^^89^ Moreover, rates of cigarette smoking in men have been much higher in Japanese.^59^^,^^87^

Three hypotheses could account for the lower CHD mortality in Japanese as compared with whites: (1) low levels of atherosclerosis in the coronary arteries of Japanese, (2) lower amounts of vulnerable plaque in the coronary arteries of Japanese, and (3) lower prevalence of risk factors related to thrombosis and clinical events. The ERA JUMP Study was initiated to test the first hypothesis.

### Major findings of the ERA JUMP Study

The ERA JUMP Study at baseline found that: (1) Japanese in Japan have significantly lower levels of atherosclerosis in the coronary and carotid arteries than whites or Japanese Americans, whereas Japanese Americans have similar or higher levels of atherosclerosis as compared with whites,^41^^,^^50^ (2) the relationship of traditional risk factors to atherosclerosis is similar across populations,^41^^,^^50^ and (3) the difference between traditional and some novel risk factors does not explain the difference in atherosclerosis between Japanese living in Japan and US populations.^90^^–^^98^ The results strongly suggest that it is unlikely that differences in atherosclerosis between Japan and the United States are a function primarily of genetic differences or genetic responses to various lifestyle factors. The most likely hypothesis is that there is a common source exposure among Japanese in Japan. The large difference in diet seemed to be the strongest candidate to explain this difference, with consumption of fish and soy showing the largest differences. Although numerous studies have examined marine-derived n-3 fatty acids and CHD^99^ since the pioneering work by Bang and Dyerberg, who observed low CHD rates among Greenland Eskimos in the 1970s,^100^ no previous studies have compared the association of marine n-3 fatty acids with atherosclerosis in Japan and other countries. Therefore, we first evaluated serum marine n-3 fatty acids and their association with subclinical atherosclerosis.

Fish consumption in Japan is among the highest in the world. The mean intake of fish is about 100 g/day in Japan, as compared with 7 g/day in the United States.^36^^,^^101^ We have found that: (1) serum levels of marine n-3 fatty acids are more than 100% higher in Japanese than in whites and Japanese Americans, (2) in each population, serum marine n-3 fatty acids had an inverse association with subclinical atherosclerosis, and (3) higher levels of marine n-3 fatty acids in Japanese in Japan significantly contributed to differences in levels of subclinical atherosclerosis between Japanese in Japan and whites.^7^^,^^102^

### Other findings

The ERA JUMP Study reported other important findings related to: (1) the association of alcohol consumption and cholesteryl ester transfer protein (CETP) in Japanese, (2) the association of serum polyunsaturated fat with cardiovascular risk factors among whites, Japanese, Japanese Americans, and Koreans, and (3) a difference in the association of obesity-related measures and biomarkers between whites and Japanese. Some topics in the ERA-JUMP Study were only examined among Japanese in Japan,^103^^–^^106^ including the associations of CAC with alcohol consumption and serum levels of CETP. We found that Japanese had a J-shaped association of alcohol consumption with CAC^103^; and that individuals with an alcohol consumption of 1 to 22 g/day had the lowest prevalence of CAC, whereas those with alcohol consumption of 69 g/day or more had the highest prevalence of CAC. We also found that serum CETP levels were positively associated with CAC.^104^ CETP plays a key role in reverse-cholesterol transport from tissue to liver. Furthermore, a genetic variant of CETP, the D442G missense mutation, is highly prevalent in Japanese (5%–10% of the general population) but very rare in whites. As compared with the lowest quartile of serum CETP, the multivariate-adjusted odds ratio for CAC was 0.77 (95% CI, 0.18 to 3.36), 0.96 (0.27 to 3.40), and 3.49 (1.05 to 11.6) for increasing CETP quartiles. These findings remained significant after additional adjustment for the CETP D442G gene variant, which has a prevalence of 5.6% in the surveyed population.

The ERA JUMP Study reported that serum levels of n-3 and n-6 fatty acids were significantly inversely associated with triglycerides and significantly and positively associated with HDL-C among whites, Japanese, and Japanese Americans.^107^ The study further reported that serum levels of linoleic and arachidonic fatty acids—2 major n-6 fatty acids—had significant inverse associations with total LDL particles and large very-low-density lipoprotein particles and significant positive associations with large HDL particles in whites, Japanese, Japanese Americans, and Koreans.^53^ Moreover, the study recently reported that serum levels of n-6 fatty acids had a significant inverse association with plasminogen-activator inhibitor-1 (PAI-1) in whites, Japanese, and Japanese Americans.^108^ These results suggest that n-6 fatty acids may be protective against CHD through lipids and PAI-1.

As compared with whites, obesity is related to cardiovascular risk clustering at a lower BMI in Japanese and other Asians.^109^ This is consistent with the results of the ERA JUMP Study, which showed differences in abdominal fat distribution and liver fat accumulation evaluated by CT and blood levels of adiponectin, PAI-1 and ghrelin. As compared with whites, Japanese had significantly more visceral adipose tissue at similar waist circumferences.^90^ Additionally, despite a lower BMI,^94^ Japanese had significantly higher levels of fat accumulation in the liver,^95^ significantly lower levels of adiponectin,^91^ significantly higher levels of PAI-1,^92^ and significantly lower levels of ghrelin.

### Future direction of the ERA JUMP Study

The follow-up study of the ERA JUMP Study started in 2007 and is ongoing. Primary hypotheses of the follow-up study are: (1) progression of atherosclerosis is slower among Japanese in Japan than in whites, (2) baseline levels of serum marine n-3 fatty acids are inversely associated with progression of atherosclerosis, and (3) higher levels of marine n-3 fatty acids in Japanese, as compared with whites significantly contribute to the difference in atherosclerosis progression. Additionally, the study is investigating the association of serum marine n-3 fatty acids with atherosclerosis in a population-based sample of 1000 Japanese, which we will describe in the next section. Furthermore, the study proposed to examine the association of phospholipid marine n-3 fatty acids with dietary data from the International Collaborative Study of Macronutrients, Micronutrients and Blood Pressure (INTERMAP) study.^110^^,^^111^

### Shiga Epidemiological Study of Subclinical Atherosclerosis (SESSA), an extension of the ERA JUMP Study in Japan

Ueshima and colleagues recruited and studied Japanese adults aged 40 to 79 years who were randomly selected from the same area (Kusatsu City, Shiga) during 2005–2008. This successive study is named the “Shiga Epidemiological Study of Subclinical Atherosclerosis (SESSA).” The method used to measure CAC in SESSA was the same as that in the original ERA JUMP Study.^7^ For the purpose of future comparison, the researchers examined whether CCS calculated in SESSA (SESSA protocol) was comparable to scores obtained by the method used in the MESA protocol.^112^ A total of 99 sets of duplicate images obtained in SESSA were sent to the MESA CT center at Harbor-UCLA medical Center.^113^ The images were scored in accordance with the MESA protocol by a MESA-certified physician, who was blinded to the original score. There was no statistically significant difference between the 2 readings (*P* = 0.79). Interclass correlation coefficients (ICCs) were similarly high regardless of whether EBCT (0.96) or MDCT (0.95) was used. The overall ICC was 0.95. Even among a subgroup of participants whose CAC score was 0 to 100 (*n* = 52), ICC was as high as 0.83. Ueshima et al are currently preparing to directly compare CAC prevalence between the participants in MESA and SESSA.

## CONCLUSIONS

The Seven Countries Study^114^ conducted by Keys et al in the late 1950s found a positive relationship between diet (especially saturated fat), serum total cholesterol, and CHD. That study was the first international epidemiologic study of CHD in the world and proved Keys’ diet-lipid-CHD hypothesis. The ERA JUMP Study, though it is much smaller than the Seven Countries Study, is a unique international population-based study of subclinical atherosclerosis, including CAC and IMT. Morbidity and mortality from CHD remain much lower in Japan than in Western countries,^115^ although fat intake and serum total cholesterol levels have increased substantially in Japan. Since epidemiologic studies and clinical trials in Japan show that traditional risk factors for CHD in Western countries are also risks for CHD in Japan,^115^ it becomes an interesting question why CHD morbidity and mortality continue to be lower in Japan than in Western countries.^116^ To address this question, the ERA JUMP Study was launched and focused on Japan and American men aged 40 to 49 with similar levels of serum total cholesterol. Although we could not evaluate the incidence rate of CHD among relatively young adults due to the low rate of CHD, we were able to compare levels of subclinical atherosclerosis across populations. The ERA JUMP Study found that, as expected, Japanese Americans living in Hawaii had a higher prevalence of CAC than did Japanese living in Japan.^41^ We cannot fully explain why Japanese in Japan had less subclinical atherosclerosis as compared with US whites, despite the similar or less favorable profile of traditional risk factors. Some potential factors responsible for the low CHD rates in Japan include the Japanese diet and its related nutrients (including fish intake, marine n-3 fatty acids, and soybeans) and obesity-years. The search to explain this observation will continue not only in the ERA JUMP but also in the SESSA, an extended study of ERA JUMP. Unknown new risk factors and preventive measures for coronary atherosclerosis may be found in the future.

Although CAC has been suggested for the screening of asymptomatic individuals at intermediate risk in Europe and the United States,^1^^,^^5^ it remains uncertain whether CAC is useful as a screening tool for asymptomatic individuals in Japan. Nevertheless, the ERA JUMP and other studies have clearly demonstrated that CAC is useful as a research tool, as it describes differences in atherosclerotic burden across populations and could thus lead to better CHD prevention.
